# The *PVT1* lncRNA is a novel epigenetic enhancer of *MYC,* and a promising risk-stratification biomarker in colorectal cancer

**DOI:** 10.1186/s12943-020-01277-4

**Published:** 2020-11-05

**Authors:** Kunitoshi Shigeyasu, Shusuke Toden, Tsuyoshi Ozawa, Takatoshi Matsuyama, Takeshi Nagasaka, Toshiaki Ishikawa, Debashis Sahoo, Pradipta Ghosh, Hiroyuki Uetake, Toshiyoshi Fujiwara, Ajay Goel

**Affiliations:** 1 Center for Gastrointestinal Research, Center for Translational Genomics and Oncology, Baylor Scott & White Research Institute and Charles A Sammons Cancer Center, Baylor University Medical Center, Dallas, TX USA; 2 Department of Gastroenterological Surgery, Okayama University Graduate School of Medicine, Dentistry, and Pharmaceutical Sciences, Okayama, Japan; 3 Department of Surgical Oncology, Graduate School of Medicine, The University of Tokyo, Tokyo, Japan; 4 Department of Specialized Surgery, Tokyo Medical and Dental University Graduate School of Medicine, Tokyo, Japan; 5 Departments of Pediatrics and Computer Science and Engineering, University of California San Diego, La Jolla, CA USA; 6 Departments of Medicine and Cellular and Molecular Medicine, University of California San Diego, La Jolla, CA USA; 7 Department of Molecular Diagnostics and Experimental Therapeutics, Beckman Research Institute of City of Hope Comprehensive Cancer Center, 1218 S. Fifth Avenue, Suite 2226, Duarte, CA USA

**Keywords:** *PVT1*, MYC, Enhancer, Epigenetic, Prognostic marker, Colorectal cancer

## Abstract

Accumulating evidence suggests that dysregulation of transcriptional enhancers plays a significant role in cancer pathogenesis. Herein, we performed a genome-wide discovery of enhancer elements in colorectal cancer (CRC). We identified *PVT1* locus as a previously unrecognized transcriptional regulator in CRC with a significantly high enhancer activity, which ultimately was responsible for regulating the expression of *MYC* oncogene. High expression of the *PVT1* long-non-coding RNA (lncRNA) transcribed from the *PVT1* locus was associated with poor survival among patients with stage II and III CRCs (*p* < 0.05). Aberrant methylation of the *PVT1* locus inversely correlated with the reduced expression of the corresponding the *PVT1* lncRNA, as well as *MYC* gene expression. Bioinformatic analyses of CRC-transcriptomes revealed that the *PVT1* locus may also broadly impact the expression and function of other key genes within two key CRC-associated signaling pathways – the TGFβ/SMAD and Wnt/β-Catenin pathways. We conclude that the *PVT1* is a novel oncogenic enhancer of *MYC* and its activity is controlled through epigenetic regulation mediated through aberrant methylation in CRC. Our findings also suggest that the *PVT1* lncRNA expression is a promising prognostic biomarker and a potential therapeutic target in CRC.

**Supplementary Information:**

The online version contains supplementary material available at 10.1186/s12943-020-01277-4.

## Main text

Accumulating evidence indicates that the pathogenesis of colorectal cancer (CRC) is influenced by epigenetic modifications. Among these, in the past decade, alterations in enhancer elements have garnered a significant attention [1], and are emerging as important players in cancer pathogenesis and being exploited as potential therapeutic targets. Recently, the FANTOM5 (Functional Annotation of the Mammalian Genome 5) project curated a genome-wide enhancer element atlas from normal tissues and tumor cells, using the cap analysis gene expression (CAGE) and next-generation sequencing (NGS) approaches [2–5]. Herein, we systematically examined the FANTOM5 database and identified the *PVT1* locus as an epigenetic enhancer in CRC and provided a novel evidence for its role in specific targeting of the *MYC* oncogene. Furthermore, we found that *PVT1* lncRNA was transcribed by the *PVT1* locus via an enhancer-like activity. Multiple in silico datasets were utilized to evaluate the clinical significance of the *PVT1* lncRNA. Pooling of seven such datasets improved the overall statistical power of the analysis and minimized potential cohort bias. Our analyses indicated that PVT1 is associated with cancer stemness and modulates key CRC-associated signaling pathways – the TGFβ/SMAD and Wnt/β-Catenin pathways. We additionally noted that high expression of the *PVT1* lncRNA associated with poorer survival in CRC patients. Taken together, our data provide first evidence that the *PVT1* locus plays a key role in CRC pathogenesis, and that it may serve as a prognostic biomarker and a potential therapeutic target in patients with CRC.

### A novel enhancer, the *PVT1* lncRNA*,* is frequently activated in colorectal cancers, and activates its enhancer potential via oncogenic *MYC*

**Fig. 1:**
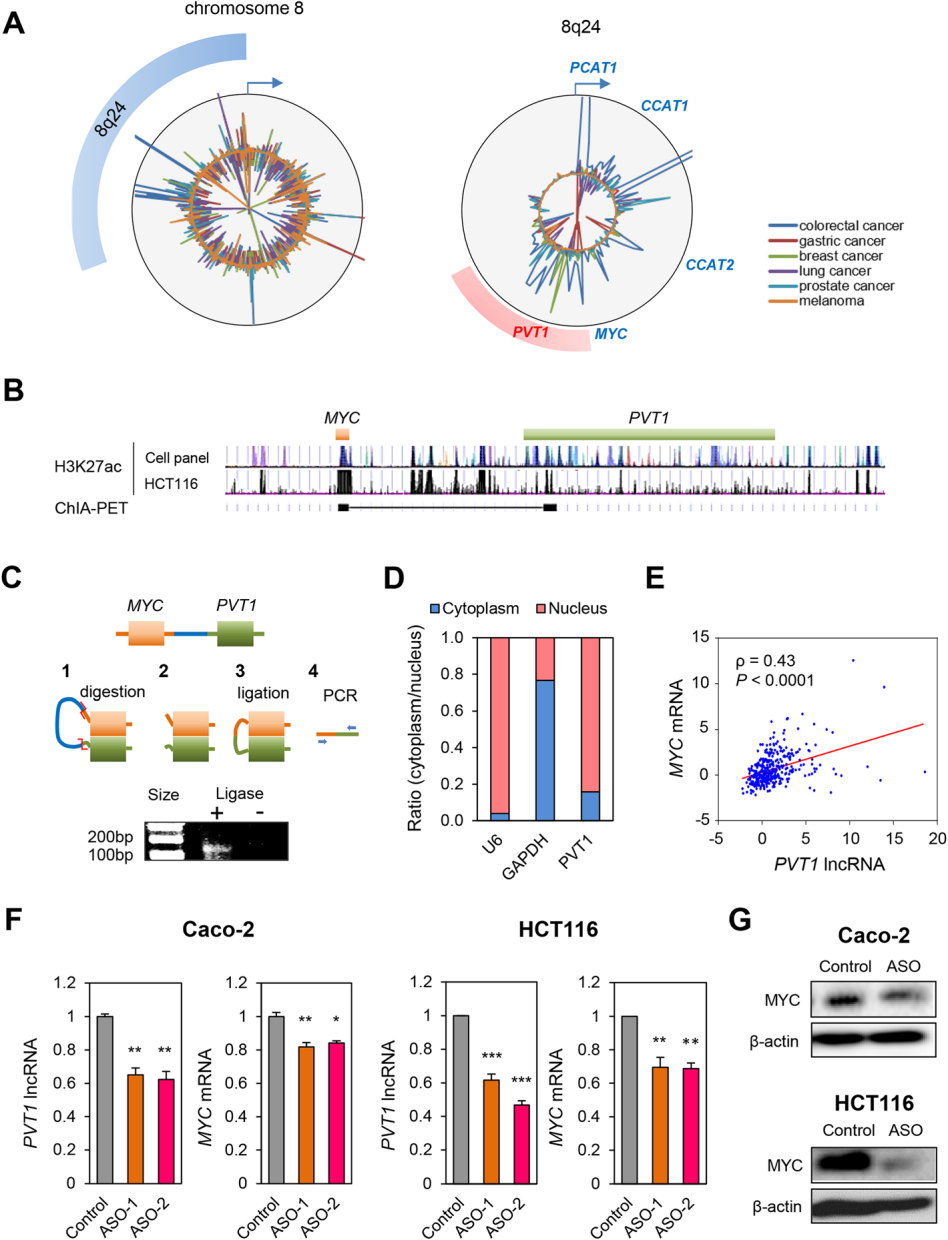
The *PVT1* locus has an enhancer element that targets *MYC*. **a** Enhancer activity within chromosome 8, and specifically within the 8q24 region, among six different cancer types (Colorectal cancer: Gastric cancer, Breast cancer, Lung cancer, Prostate cancer, and Melanoma) according to the FANTOM5 database. **b** Comparison panel of two different analyses; H3K27ac signal in a cell line panel (GM12878, H1-hESC, HSMM, HUVEC, K562, NHEK, NHLF) and in HCT116 (CRC cell line); Chia-PET in K562 leukemia cell line (as revealed by exploring the UCSC genome browser). **c** 3C assay to detect the *PVT1*-*MYC* interaction in HCT116 cells. **d** RT-qPCR determination of the *PVT1* lncRNA, U6 snRNA (nucleus-specific), and GAPDH mRNA (cytoplasm-specific) levels in nuclear and cytosolic extracts from HCT116 cells. **e** Plot for the *PVT1* lncRNA and *MYC* mRNA expression levels in CRC. **f** Effect of knockdown of the *PVT1* lncRNA on expression levels of the *PVT1* lncRNA and *MYC* mRNA in Caco-2 and HCT116 cells as determined by RT-qPCR. **g** Western immunoblotting to determine the effect of knockdown of the *PVT1* lncRNA on MYC protein levels in Caco-2 and HCT116 cells. *P < 0.05, **P < 0.01, ***P < 0.001

We performed a systematic analysis of 43,011 enhancer elements that were recently reported in the FANTOM5 enhancer database (Supplementary Table 1). We observed that the strongest enhancer activity in CRCs was confined to the classic, CRC-associated chromosome 8q24 region – often referred to as the ‘gene desert’, including the genes such as the *PCAT1*, *CCAT1, CCAT2, PVT1* and *MYC* (Fig. 1a). In particular, *CCAT1-L* lncRNA, which is transcribed from the *CCAT1* locus, has been shown to stabilize loop structure between *CCAT1* and *MYC* via an “enhancer-like function” [6, 7]. Therefore, we were intrigued by the identification of a novel locus, *PVT1*, which has previously not been reported as an enhancer region in CRC. In the genome-wide FANTOM5 enhancer database, the *PVT1* locus exhibited cancer-specific enhancer activity, especially in CRCs. We analyzed this region carefully using the University of California Santa Cruz (UCSC) genome browser. We discovered that the *PVT1* locus indeed exhibited a strong H3K27ac enhancer signal, in a panel of cell lines (GM12878, H1-hESC, HSMM, HUVEC, K562, NHEK and NHLF), as well as in HCT116 CRC cells (Fig. 1b). Next, we analyzed Chromatin Interaction Analysis by Paired-End Tag Sequencing (ChIA-PET) results from the UCSC genome browser and demonstrated that the *PVT1* region interacts with the *MYC* oncogene (Fig. 1b).

Our results from the FANTOM5 database and the UCSC genome browser analysis lead to the hypothesis that the *PVT1* region might have oncogenic enhancer activity that targets the *MYC* oncogene in CRC cells. Using a chromosome conformation capture (3C) assay in HCT116 cell lysates, we demonstrated that indeed the *PVT1* locus formed a loop structure in a *cis* conformation with *MYC* (Fig. 1c).

We next measured the ability of the *PVT1* lncRNA for its ability to drive the expression of *MYC* in CRC cells. Recent reports suggest that enhancers may produce lncRNAs that can stabilize a *cis* conformation between enhancers and promoters [7]. This mechanism of enhancer-related lncRNA activity primarily occurs inside the nucleus*.* We found that majority of the *PVT1* lncRNA was indeed present within the nuclear compartment (Fig. 1d). In addition, we were very encouraged to observe that a positive correlation between this noncoding RNA and the *MYC* gene in the CRC specimens (cohort 1, *ρ =* 0.43, *P* < 0.0001, Fig. 1e).

To further investigate the role for the *PVT1’s* enhancer activity, we next performed knockdown experiments for the *PVT1* lncRNA. Although the *PVT1* lncRNA knockdown using siRNA has already been previously attempted, such efforts did not result in concomitant suppression of the *MYC* mRNA [8], because establishing an effective nuclear *PVT1* lncRNA knockdown using siRNAs is challenging. To overcome this issue, we established a specific antisense oligonucleotide (ASO) that targets the *PVT1* lncRNA and permits its knockdown in the nucleus. Our approach led to successful knockdown of the *PVT1* lncRNA in the cancer cell lines (*P* < 0.01 in Caco-2, *P* < 0.001 in HCT116), with a simultaneous transcriptional suppression of the *MYC* mRNA (*P* < 0.05 in Caco-2, P < 0.01 in HCT116, Fig. 1f) and MYC protein levels (Fig. 1g), suggesting an enhancer-like function.

### The *PVT1* lncRNA is frequently overexpressed in stage II and III CRCs

Next, we analyzed the *PVT1* lncRNA expression in CRC specimens. We evaluated the expression levels of the *PVT1* lncRNA in stage II and III CRCs. In multivariate analysis, high-*PVT1* expression emerged as an independent prognostic factor in stage II and III CRC patients (Cohort-1: *P* = 0.0246; Cohort-2: *P* = 0.0196, Supplementary Table 2 and 3). Data derived from these clinical cohorts further highlight that the *PVT1* lncRNA expression levels may serve as an important prognostic biomarker for stage II and III CRC patients, and can facilitate stratification of appropriate patient subsets that are optimal candidates for benefitting from adjuvant chemotherapy and attenuate recurrence [9].

### *PVT1* lncRNA expression increases in CRC metastases, and its expression is widely associated with genes within the TGFβ/SMAD and Wnt/β-catenin pathways

**Fig. 2:**
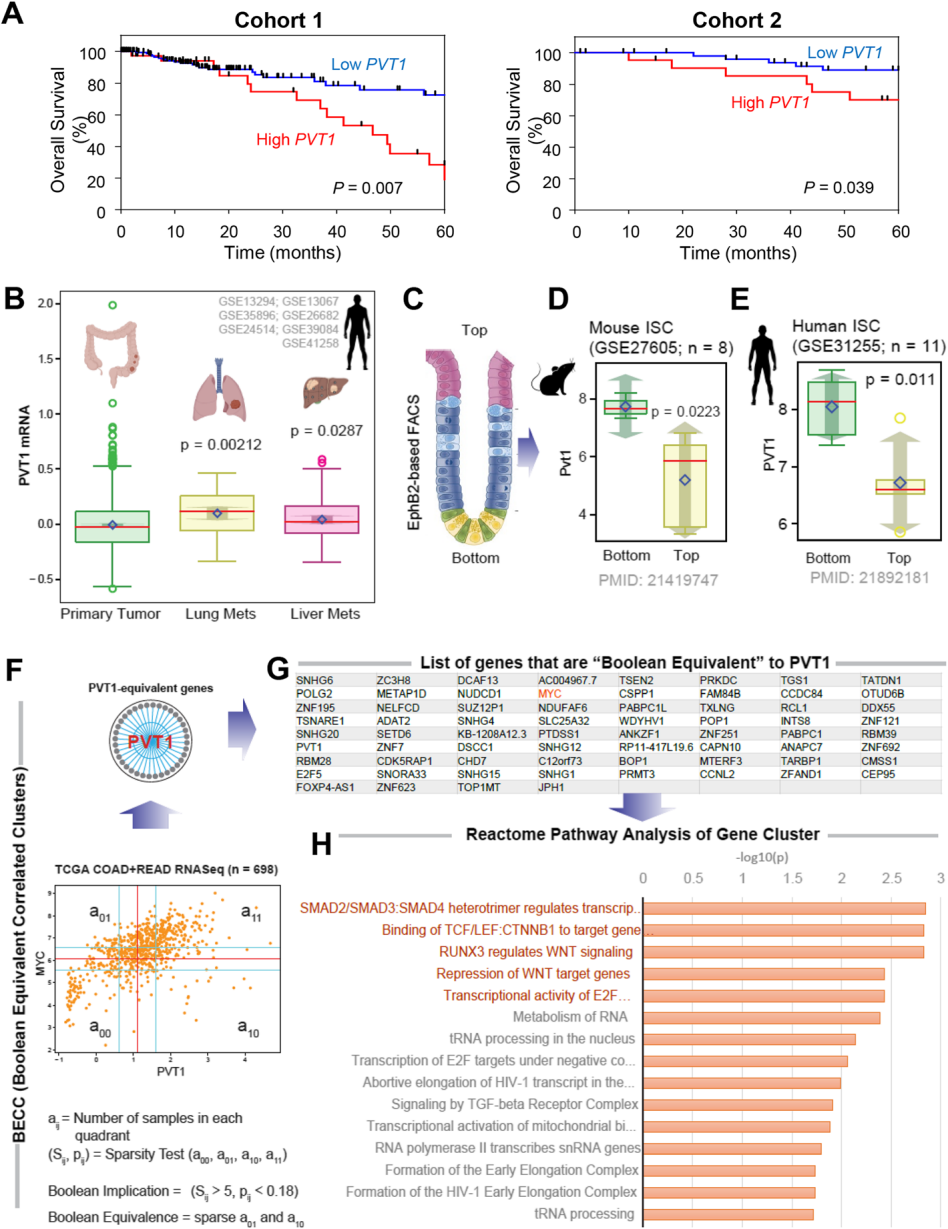
The *PVT1* lncRNA is overexpressed in CRC. *PVT1* lncRNA is highly expressed in CRC metastases and within crypt bases; is associated within gene clusters that regulate the TGFβ/SMAD2/3/4 and Wnt/β-Catenin pathways. **a** Overall Survival plot for patients with high-*PVT1* lncRNA expression versus patients with low-*PVT1* lncRNA expression in the two cohorts (*P* < 0.007 in Cohort-1, *P* < 0.039 in Cohort-2). **b** Whisker plots showing the levels of expression of *PVT1* lncRNA in CRCs (primary and metastases) from 7 pooled datasets. **c-e** Schematic in C shows the EphB2-based FACS analyses approach used to separate epithelial cells at the bottom of the crypt from those at the top. Mouse (**d**) and human (**e**) datasets show whisker plots of the levels of expression of *PVT1* lncRNA at the bottom and top of the crypts. **f** Computational approach to identify clusters of genes that share Boolean Equivalent relationships between each other, in this case identified using the *PVT1* lncRNA as ‘seed’ in TCGA COAD dataset (*n* = 698) (top panel). Number of samples in all four quadrants are used to compute two parameters (S, p). S > 5 and *p* < 0.05 is used to identify sparse quadrant. Equivalent relationships are discovered when top-left and bottom-right quadrants are sparse (lower panel). **g** List of genes that are equivalent to the *PVT1* lncRNA. MYC is highlighted in red. **h** Reactome pathway analysis shows pathways that are most prominently enriched (highlighted in red) in the *PVT1*-equivalent cluster

We next evaluated the expression pattern of the *PVT1* lncRNA, and the related functional pathways to clarify its clinical significance in data gathered from 7 pooled datasets (*see* *methods*). The expression levels of the *PVT1* lncRNA were significantly higher in both lung and liver metastases compared with the primary lesions (Fig. 2b). Intriguingly, the *PVT1* lncRNA was overexpressed at the bottom of the colorectal crypt compared with the top, both in mice (*P* = 0.022) and humans (*P* = 0.011); suggesting that *PVT1* lncRNA may either favor or serve as a marker of stemness which exists at the bottom of the crypt (Fig. 2c-e). Together, these findings using unbiased bioinformatic approaches suggest that the *PVT1* lncRNA may promote distant metastasis, perhaps via its ability to promote stemness in the colon cells.

Finally, we asked what other genes may be impacted by the *PVT1* lncRNA, or vice versa. To this end, we analyzed The Cancer Genome Atlas (TCGA) CRC datasets (*n* = 698; Fig. 2f) using Boolean equivalent correlated clusters (BECC) analysis [10]. We used the *PVT1* lncRNA as a seed gene and identified a set of 67 genes (Fig. 2g) that displayed a tight, statistically significant Boolean Equivalent relationship to the *PVT1* lncRNA across all 698 CRCs in the dataset, as determined by BooleanNet statistics; and indeed *MYC* appeared as one of the key genes even in these analysis (Fig. 2f-g). The Reactome pathway analyses revealed that most of the 67 genes served within two major signaling pathways, i.e., TGFβ/SMAD2/3/4 and Wnt/β-Catenin pathways (Fig. 2h). These findings further support our hypothesis that the *PVT1* lncRNA widely impacts major signaling pathways in CRC by regulating the expression of key genes, such as the *MYC*.

### The *PVT1* locus is epigenetically regulated and its methylation status inversely correlates with its transcriptional levels in CRC

**Fig. 3:**
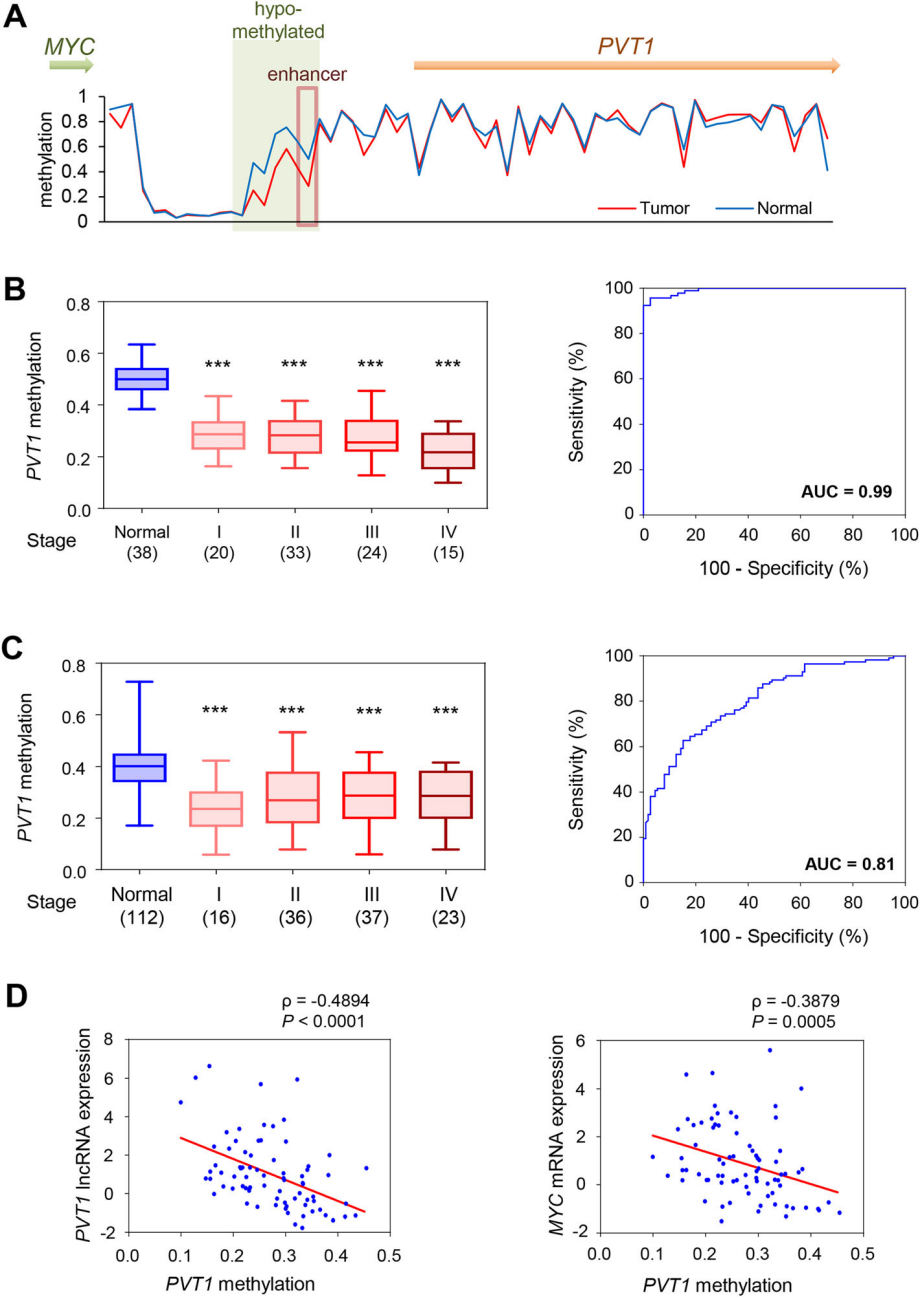
The *PVT1* locus is hypomethylated and methylation state negatively correlated with its lncRNA expression in CRC. **a** The methylation levels at the *PVT1* locus in CRC and normal mucosa determined using a 450 K methylation array derived from TCGA database. **b** Methylation levels at the CpG site (cg23898497) in CRC compared to normal mucosa in all stages in Cohort-1 (*P* < 0.001, AUC = 0.99) according to the FANTOM5 database. **c** Methylation levels at the CpG site (cg23898497) in CRC compared to normal mucosa in all stages in Cohort-3 (*P <* 0.001, AUC = 0.81) as determined by pyrosequencing. **d** Plot of the *PVT1* methylation versus its lncRNA expression (*ρ =* − 0.4894, *P <* 0.0001), and the *PVT1* methylation versus *MYC* expression (*ρ =* − 0.3879, *P* = 0.0005) in Cohort-1. ***P < 0.001

Aberrant methylation is one of the key regulators of enhancer activity in various genes. Interestingly, we observed a significant loss in CpG sequence methylation in the vicinity of the *PVT1* locus, including its enhancer cluster (Fig. 3a). Specifically, a CpG site (cg23898497) in the middle of its enhancer region (chr8:128822251–128,823,013) was significantly hypomethylated in CRC vs. normal mucosa, in all disease stages in the cohort-1 (*P* < 0.001, area under the curve: AUC = 0.99, Fig. 3b) and cohort-3 patients (*P <* 0.001, AUC = 0.81, Fig. 3c). These results suggest that the *PVT1* enhancer activity and the expression of this lncRNA might be controlled through an epigenetic regulation of this region. In support of our other findings, the methylation status of the *PVT1* region negatively correlated with its lncRNA expression (*ρ =* − 0.4894, *P <* 0.0001), as well as with the *MYC* gene expression (*ρ =* − 0.3879, *P* = 0.0005, Fig. 3d).

## Conclusions

Previously, the *PVT1* lncRNA was reported as a stabilizer of the MYC protein [8]. In addition, our study indicates that the *PVT1* locus may directly controls the *MYC* mRNA expression as an enhancer. Based on these data, targeting of the *PVT1* lncRNA may be a potential therapeutic approach in CRC patients, which could eventually lead to suppression of the oncogenic *MYC*, at both the RNA and protein levels.

## Supplementary Information

**Additional file 1: Supplementary Table 1**

**Additional file 2: Supplementary Table 2–7**

**Additional file 3: Methods**

## Data Availability

All data derived from public database are available from these sites. FANTOM5_Human_Enhancer_Tracks: http://slidebase.binf.ku.dk/human_enhancers/presets TCGA_Research_Network: http://cancergenome.nih.gov/ cBioPortal: http://www.cbioportal.org/index.do UCSC_Genome_Browser: http://genome.ucsc.edu/ All other data are contained within this article.

## Abbreviations

CRC: Colorectal cancer
FANTOM5: Functional Annotation of the Mammalian Genome 5
CAGE: Cap analysis gene expression
NGS: Next-generation sequencing
UCSC: University of California, Santa Cruz
ChIA-PET: Chromatin Interaction Analysis by Paired-End Tag Sequencing
3C: Chromosome conformation capture
RISC: RNA-induced silencing complex
ASO: Antisense oligonucleotide
TCGA: The Cancer Genome Atlas
BECC: Boolean equivalent correlated clusters
