# Supplementary material for: The PVT1 lncRNA is a novel epigenetic enhancer of MYC, and a promising risk-stratification biomarker in colorectal cancer
Source: Mol Cancer. 2020 Nov 5;19:155. doi: 10.1186/s12943-020-01277-4 (PMC7643275; doi:10.1186/s12943-020-01277-4)
Supplement: Supplementary file 2 — Additional file 2: Supplementary Table 2–7 [file 12943_2020_1277_MOESM2_ESM.docx]

**Supplementary Data 2**

**Supplementary Table 2:** Univariate and multivariate analysis of the predictors of OS in patients with stage II and III CRC (Cohort-1).

|  |  |  | Univariate | | |  | Multivariate | | |  |
| --- | --- | --- | --- | --- | --- | --- | --- | --- | --- | --- |
|  | Characteristics |  | HR | 95% CI | *P* |  | HR | 95% CI | *P* |  |
|  | Gender |  |  |  |  |  |  |  |  |  |
|  | Male/Female |  | 1.00 | 0.54-1.85 | 0.9912 |  | 0.91 | 0.48-1.74 | 0.7739 |  |
|  | Age |  |  |  |  |  |  |  |  |  |
|  | >70/<70 years old |  | **2.37** | **1.26-4.64** | **0.0068** |  | **2.32** | **1.22-4.62** | **0.0101** |  |
|  | Location |  |  |  |  |  |  |  |  |  |
|  | Colon/Rectum |  | 0.81 | 0.38-2.02 | 0.6328 |  | 1.54 | 0.60-4.35 | 0.3794 |  |
|  | Stage |  |  |  |  |  |  |  |  |  |
|  | II/III |  | 1.50 | 0.81-2.79 | 0.1964 |  | 1.62 | 0.86-3.07 | 0.1348 |  |
|  | *PVT1* lncRNA |  |  |  |  |  |  |  |  |  |
|  | High/Low |  | **2.36** | **1.21-4.42** | **0.0126** |  | **2.40** | **1.12-4.89** | **0.0246** |  |

**Supplementary Table 3:** Univariate and multivariate analysis of the predictors of OS in patients with stage II and III CRC (Cohort-2).

|  |  |  | Univariate | | |  | Multivariate | | |  |
| --- | --- | --- | --- | --- | --- | --- | --- | --- | --- | --- |
|  | Characteristics |  | HR | 95% CI | *P* |  | HR | 95% CI | *P* |  |
|  | Gender |  |  |  |  |  |  |  |  |  |
|  | Male/Female |  | 0.81 | 0.26-2.45 | 0.7099 |  | 1.12 | 0.36-3.42 | 0.8406 |  |
|  | Age |  |  |  |  |  |  |  |  |  |
|  | >65/<65 years old |  | 1.81 | 0.55-8.08 | 0.3437 |  | 1.52 | 0.45-6.87 | 0.5204 |  |
|  | Location |  |  |  |  |  |  |  |  |  |
|  | Colon/Rectum |  | 0.75 | 0.24-2.26 | 0.6045 |  | 0.82 | 0.25-2.61 | 0.7314 |  |
|  | Stage |  |  |  |  |  |  |  |  |  |
|  | II/III |  | **4.78** | **1.46-21.34** | **0.0086** |  | **5.66** | **1.65-26.07** | **0.0050** |  |
|  | *PVT1* lncRNA |  |  |  |  |  |  |  |  |  |
|  | High /Low |  | **2.99** | **0.99-9.29** | **0.0520** |  | **4.00** | **1.26-12.99** | **0.0196** |  |

**Supplementary Table 4:** Clinicopathological features of patients in Cohort 1 and 2.

|  | **Characteristics** | **Cohort 1** | **Cohort 2** |
| --- | --- | --- | --- |
|  | **No. of patients** | 239 | 75 |
|  | **Sex (Male/Female)** | 127/ 112 | 35/ 40 |
|  | **Age (Mean)** | 65.4 | 69.3 |
|  | **Location (Colon/Rectum)** | 180/ 59 | 42/ 33 |
|  | **Tumor depth (T1/2/3/4)** | 1/ 9/ 206/ 23 | 0/ 5/ 39/ 31 |
|  | **Lymph node meta (negative/positive)** | 127/ 112 | 44/ 31 |

**Supplementary Table 5:** Primer sequences for PCR.

| **Genes** | **Primer sequences** |
| --- | --- |
| *PVT1* | Forward: TTGGGTCTCCCTATGGAATG |
|  | Reverse: GGAGAAGGCTCCAGGGAGTA |
| *MYC* | Forward: CGTCTCCACACATCAGCACAA |
|  | Reverse: TCTTGGCAGCAGGATAGTCCTT |
| *β-actin* | Forward: AGAGCTACGAGCTGCCTGAC |
|  | Reverse: AGCACTGTGTTGGCGTACAG |
| 3C assay  between *PVT1* and *MYC* | Forward: tcccctgctcactccaaac |
|  | Reverse: caggcccctatttctttgttct |
| *PVT1* methylation analysis | Forward: aggagggaggtgTaggatgTtT |
|  | Reverse: (biotin): ccccaAAccttAcaaaAAcca |
|  | Sequence: GTAGGATGTTTTTTGGT |

**Supplementary Table 6:** Antisense oligonucleotide sequence.

| **Targets** | **Sequences** |
| --- | --- |
| *PVT1* ASO-1 | mU*mA*mA*mU*mG*G*G*T*T*G*A*A*G*C*T*mG*mU*mC*mC*mU |
| *PVT1* ASO-2 | mG*mU*mG*mG*mA*C*A*G*G*T*A*A*C*A*G*mG*mU*mG*mC*mU |
| Control ASO | mG*mC*mG*mU*mA*T*T*A*T*A*G*C*C*G*A*mU*mU*mA*mA*mC |

**Supplementary Table 7:** Table of Reactomes (Complete).

| Name | pValue | FDR |
| --- | --- | --- |
| [SMAD2/SMAD3:SMAD4 heterotrimer regulates transcription](http://www.reactome.org/PathwayBrowser/#/R-HSA-2173796&DTAB=AN&ANALYSIS=MjAyMDA0MDcxODMwNTNfMTEzMA%3D%3D) | 0.001415483885423563 | 0.1498182676921691 |
| [Binding of TCF/LEF:CTNNB1 to target gene promoters](http://www.reactome.org/PathwayBrowser/#/R-HSA-4411364&DTAB=AN&ANALYSIS=MjAyMDA0MDcxODMwNTNfMTEzMA%3D%3D) | 0.0014833491850709812 | 0.1498182676921691 |
| [RUNX3 regulates WNT signaling](http://www.reactome.org/PathwayBrowser/#/R-HSA-8951430&DTAB=AN&ANALYSIS=MjAyMDA0MDcxODMwNTNfMTEzMA%3D%3D) | 0.0014833491850709812 | 0.1498182676921691 |
| [Repression of WNT target genes](http://www.reactome.org/PathwayBrowser/#/R-HSA-4641265&DTAB=AN&ANALYSIS=MjAyMDA0MDcxODMwNTNfMTEzMA%3D%3D) | 0.0037160164199128154 | 0.2049695044876787 |
| [Transcriptional activity of SMAD2/SMAD3:SMAD4 heterotrimer](http://www.reactome.org/PathwayBrowser/#/R-HSA-2173793&DTAB=AN&ANALYSIS=MjAyMDA0MDcxODMwNTNfMTEzMA%3D%3D) | 0.0037246255509812976 | 0.2049695044876787 |
| [Metabolism of RNA](http://www.reactome.org/PathwayBrowser/#/R-HSA-8953854&DTAB=AN&ANALYSIS=MjAyMDA0MDcxODMwNTNfMTEzMA%3D%3D) | 0.004099390089753574 | 0.2049695044876787 |
| [tRNA processing in the nucleus](http://www.reactome.org/PathwayBrowser/#/R-HSA-6784531&DTAB=AN&ANALYSIS=MjAyMDA0MDcxODMwNTNfMTEzMA%3D%3D) | 0.007234329188603672 | 0.30869491384913916 |
| [Transcription of E2F targets under negative control by DREAM complex](http://www.reactome.org/PathwayBrowser/#/R-HSA-1362277&DTAB=AN&ANALYSIS=MjAyMDA0MDcxODMwNTNfMTEzMA%3D%3D) | 0.008783196621150013 | 0.30869491384913916 |
| [Abortive elongation of HIV-1 transcript in the absence of Tat](http://www.reactome.org/PathwayBrowser/#/R-HSA-167242&DTAB=AN&ANALYSIS=MjAyMDA0MDcxODMwNTNfMTEzMA%3D%3D) | 0.01017142219828071 | 0.30869491384913916 |
| [Signaling by TGF-beta Receptor Complex](http://www.reactome.org/PathwayBrowser/#/R-HSA-170834&DTAB=AN&ANALYSIS=MjAyMDA0MDcxODMwNTNfMTEzMA%3D%3D) | 0.012205716343013262 | 0.30869491384913916 |
| [Transcriptional activation of mitochondrial biogenesis](http://www.reactome.org/PathwayBrowser/#/R-HSA-2151201&DTAB=AN&ANALYSIS=MjAyMDA0MDcxODMwNTNfMTEzMA%3D%3D) | 0.012984692683171595 | 0.30869491384913916 |
| [RNA polymerase II transcribes snRNA genes](http://www.reactome.org/PathwayBrowser/#/R-HSA-6807505&DTAB=AN&ANALYSIS=MjAyMDA0MDcxODMwNTNfMTEzMA%3D%3D) | 0.015931503622043208 | 0.30869491384913916 |
| [Formation of the Early Elongation Complex](http://www.reactome.org/PathwayBrowser/#/R-HSA-113418&DTAB=AN&ANALYSIS=MjAyMDA0MDcxODMwNTNfMTEzMA%3D%3D) | 0.018429121792340464 | 0.30869491384913916 |
| [Formation of the HIV-1 Early Elongation Complex](http://www.reactome.org/PathwayBrowser/#/R-HSA-167158&DTAB=AN&ANALYSIS=MjAyMDA0MDcxODMwNTNfMTEzMA%3D%3D) | 0.018429121792340464 | 0.30869491384913916 |
| [tRNA processing](http://www.reactome.org/PathwayBrowser/#/R-HSA-72306&DTAB=AN&ANALYSIS=MjAyMDA0MDcxODMwNTNfMTEzMA%3D%3D) | 0.019177802064506144 | 0.30869491384913916 |
| [G0 and Early G1](http://www.reactome.org/PathwayBrowser/#/R-HSA-1538133&DTAB=AN&ANALYSIS=MjAyMDA0MDcxODMwNTNfMTEzMA%3D%3D) | 0.01936942370628536 | 0.30869491384913916 |
| [Tat-mediated HIV elongation arrest and recovery](http://www.reactome.org/PathwayBrowser/#/R-HSA-167243&DTAB=AN&ANALYSIS=MjAyMDA0MDcxODMwNTNfMTEzMA%3D%3D) | 0.020329546630912798 | 0.30869491384913916 |
| [Pausing and recovery of Tat-mediated HIV elongation](http://www.reactome.org/PathwayBrowser/#/R-HSA-167238&DTAB=AN&ANALYSIS=MjAyMDA0MDcxODMwNTNfMTEzMA%3D%3D) | 0.020329546630912798 | 0.30869491384913916 |
| [CREB1 phosphorylation through NMDA receptor-mediated activation of RAS signaling](http://www.reactome.org/PathwayBrowser/#/R-HSA-442742&DTAB=AN&ANALYSIS=MjAyMDA0MDcxODMwNTNfMTEzMA%3D%3D) | 0.020329546630912798 | 0.30869491384913916 |
| [HIV elongation arrest and recovery](http://www.reactome.org/PathwayBrowser/#/R-HSA-167287&DTAB=AN&ANALYSIS=MjAyMDA0MDcxODMwNTNfMTEzMA%3D%3D) | 0.021309246595429765 | 0.30869491384913916 |
| [Pausing and recovery of HIV elongation](http://www.reactome.org/PathwayBrowser/#/R-HSA-167290&DTAB=AN&ANALYSIS=MjAyMDA0MDcxODMwNTNfMTEzMA%3D%3D) | 0.021309246595429765 | 0.30869491384913916 |
| [Major pathway of rRNA processing in the nucleolus and cytosol](http://www.reactome.org/PathwayBrowser/#/R-HSA-6791226&DTAB=AN&ANALYSIS=MjAyMDA0MDcxODMwNTNfMTEzMA%3D%3D) | 0.02166666184601962 | 0.30869491384913916 |
| [Signaling by TGF-beta family members](http://www.reactome.org/PathwayBrowser/#/R-HSA-9006936&DTAB=AN&ANALYSIS=MjAyMDA0MDcxODMwNTNfMTEzMA%3D%3D) | 0.026267308323665417 | 0.30869491384913916 |
| [Transcriptional regulation by RUNX3](http://www.reactome.org/PathwayBrowser/#/R-HSA-8878159&DTAB=AN&ANALYSIS=MjAyMDA0MDcxODMwNTNfMTEzMA%3D%3D) | 0.028676980644709582 | 0.30869491384913916 |
| [rRNA processing in the nucleus and cytosol](http://www.reactome.org/PathwayBrowser/#/R-HSA-8868773&DTAB=AN&ANALYSIS=MjAyMDA0MDcxODMwNTNfMTEzMA%3D%3D) | 0.028945065718709584 | 0.30869491384913916 |
| [Formation of HIV-1 elongation complex containing HIV-1 Tat](http://www.reactome.org/PathwayBrowser/#/R-HSA-167200&DTAB=AN&ANALYSIS=MjAyMDA0MDcxODMwNTNfMTEzMA%3D%3D) | 0.030968257385895592 | 0.30869491384913916 |
| [Formation of HIV elongation complex in the absence of HIV Tat](http://www.reactome.org/PathwayBrowser/#/R-HSA-167152&DTAB=AN&ANALYSIS=MjAyMDA0MDcxODMwNTNfMTEzMA%3D%3D) | 0.03213075150812528 | 0.30869491384913916 |
| [TFAP2 (AP-2) family regulates transcription of cell cycle factors](http://www.reactome.org/PathwayBrowser/#/R-HSA-8866911&DTAB=AN&ANALYSIS=MjAyMDA0MDcxODMwNTNfMTEzMA%3D%3D) | 0.03293719201587175 | 0.30869491384913916 |
| [Tat-mediated elongation of the HIV-1 transcript](http://www.reactome.org/PathwayBrowser/#/R-HSA-167246&DTAB=AN&ANALYSIS=MjAyMDA0MDcxODMwNTNfMTEzMA%3D%3D) | 0.034506594280158565 | 0.30869491384913916 |
| [HIV Transcription Elongation](http://www.reactome.org/PathwayBrowser/#/R-HSA-167169&DTAB=AN&ANALYSIS=MjAyMDA0MDcxODMwNTNfMTEzMA%3D%3D) | 0.034506594280158565 | 0.30869491384913916 |
| [Mitochondrial biogenesis](http://www.reactome.org/PathwayBrowser/#/R-HSA-1592230&DTAB=AN&ANALYSIS=MjAyMDA0MDcxODMwNTNfMTEzMA%3D%3D) | 0.034513870296952964 | 0.30869491384913916 |
| [RMTs methylate histone arginines](http://www.reactome.org/PathwayBrowser/#/R-HSA-3214858&DTAB=AN&ANALYSIS=MjAyMDA0MDcxODMwNTNfMTEzMA%3D%3D) | 0.03571950398133816 | 0.30869491384913916 |
| [Activation of AKT2](http://www.reactome.org/PathwayBrowser/#/R-HSA-165158&DTAB=AN&ANALYSIS=MjAyMDA0MDcxODMwNTNfMTEzMA%3D%3D) | 0.03832156681340604 | 0.30869491384913916 |
| [NrCAM interactions](http://www.reactome.org/PathwayBrowser/#/R-HSA-447038&DTAB=AN&ANALYSIS=MjAyMDA0MDcxODMwNTNfMTEzMA%3D%3D) | 0.03832156681340604 | 0.30869491384913916 |
| [NOTCH1 Intracellular Domain Regulates Transcription](http://www.reactome.org/PathwayBrowser/#/R-HSA-2122947&DTAB=AN&ANALYSIS=MjAyMDA0MDcxODMwNTNfMTEzMA%3D%3D) | 0.04073269709900651 | 0.30869491384913916 |
| [Synthesis of PS](http://www.reactome.org/PathwayBrowser/#/R-HSA-1483101&DTAB=AN&ANALYSIS=MjAyMDA0MDcxODMwNTNfMTEzMA%3D%3D) | 0.043676330771468264 | 0.30869491384913916 |
| [rRNA processing](http://www.reactome.org/PathwayBrowser/#/R-HSA-72312&DTAB=AN&ANALYSIS=MjAyMDA0MDcxODMwNTNfMTEzMA%3D%3D) | 0.04859583784072019 | 0.30869491384913916 |
| [Formation of RNA Pol II elongation complex](http://www.reactome.org/PathwayBrowser/#/R-HSA-112382&DTAB=AN&ANALYSIS=MjAyMDA0MDcxODMwNTNfMTEzMA%3D%3D) | 0.04871406599178418 | 0.30869491384913916 |
