# Supplementary material for: The PVT1 lncRNA is a novel epigenetic enhancer of MYC, and a promising risk-stratification biomarker in colorectal cancer
Source: Mol Cancer. 2020 Nov 5;19:155. doi: 10.1186/s12943-020-01277-4 (PMC7643275; doi:10.1186/s12943-020-01277-4)
Supplement: Supplementary file 3 — Additional file 3: Methods [file 12943_2020_1277_MOESM3_ESM.docx]

**The *PVT1* lncRNA is a novel epigenetic enhancer of *MYC,* and a risk-stratification biomarker in colorectal cancer**

Kunitoshi Shigeyasu^1, 2^, Shusuke Toden^1^, Tsuyoshi Ozawa^1, 3^, Takatoshi Matsuyama^1, 4^, Takeshi Nagasaka^2^, Toshiaki Ishikawa^4^, Debashis Sahoo^5^, Pradipta Ghosh^6^, Hiroyuki Uetake^4^, Toshiyoshi Fujiwara^2^, and Ajay Goel^1, 7,*^

^1^ Center for Gastrointestinal Research, Center for Translational Genomics and Oncology, Baylor Scott & White Research Institute and Charles A Sammons Cancer Center, Baylor University Medical Center, Dallas, Texas, USA

^2^ Department of Gastroenterological Surgery, Okayama University Graduate School of Medicine, Dentistry, and Pharmaceutical Sciences, Okayama, Japan

^3^ Surgical Oncology & Vascular Surgery, The University of Tokyo, Tokyo, Japan

^4^Department of Surgical Oncology, Tokyo Medical and Dental University Graduate School, Tokyo, Japan

^5^ Departments of Pediatrics and Computer Science and Engineering, University of California San Diego, La Jolla, CA, USA

^6^ Departments of Medicine and Cellular and Molecular Medicine, University of California San Diego, La Jolla, CA, USA

^7^ Department of Molecular Diagnostics and Experimental Therapeutics, Beckman Research Institute of City of Hope Comprehensive Cancer Center, Duarte, CA, USA

Corresponding author: Ajay Goel, PhD, Department of Molecular Diagnostics and Experimental Therapeutics, Beckman Research Institute of City of Hope Comprehensive Center; 1218 S. Fifth Avenue, Suite 2226, Biomedical Research Center, Monrovia, CA 91016; Phone: 626-218-3452; Email: [ajgoel@coh.org](mailto:ajgoel@coh.org)

**METHODS**

Patient cohorts and specimens

This study included analysis of a total of 426 CRC patients. For the *PVT1* expression analysis, we analyzed a total of 314 colorectal tissues from two clinical cohorts (Cohort-1: 239 cases; Cohort-2: 75 cases). Cohort-1 was obtained from The Cancer Genome Atlas (TCGA) database [[1-4](#_ENREF_1)] and Cohort-2 included 75 tissue specimens from patients enrolled at the Tokyo Medical and Dental University, Tokyo, Japan. Tissues from stage II and III CRC patients were analyzed in each cohort (**Supplementary Table 4**). Additionally, for the *PVT1* methylation analysis, 112 CRC specimens obtained from the Okayama University, Okayama, Japan, were also analyzed (Cohort-3). All study-related procedures were performed as per the Declarations of Helsinki, wherein a written informed consent was obtained from each patient, and the institutional review boards of all participating institutions involved approved the study.

Enhancer analyses from the FANTOM5 database and the UCSC genome browser

Original data for the enhancer activity were downloaded from the FANTOM5 database [[5-7](#_ENREF_5)]. The relative activation of each enhancer element was determined by the formula: [(CACO2 - colon adult donor1) + (COLO320 - colon adult donor1)]/2 in CRC, [(MKN1 - stomach fetal donor1) + (MKN45 - stomach fetal donor1)]/2 in gastric cancer (GC), [(MCF7 - breast adult donor1) + (MDA-MB-453 - breast adult donor1)]/2 in breast cancer (BC), [(A549 - lung right lower lobe adult donor1) + (PC-14 - lung right lower lobe adult donor1)]/2 in lung cancer (LC), [(DU145 - Prostate Epithelial Cells (polarized) donor1) + (PC-3 - Prostate Epithelial Cells (polarized) donor1)]/2 in prostate cancer (PC), and [(COLO679 - Melanocyte light donor1) + (G361 - Melanocyte light donor1)]/2 in melanoma (**Supplementary Table 1**). Chromatin Interaction Analysis by ChIA-PET data were obtained from the UCSC genome browser [[8](#_ENREF_8), [9](#_ENREF_9)]. K562 cell data obtained from ChIA-PET were used as a positive control when performing target analysis of the *PVT1* enhancer activity.

Cell lines

The CRC cell lines, Caco-2 and HCT116 were purchased from the American Type Culture Collection (ATCC, Rockville, MD, USA). All cell lines were cultured according to the manufacturer’s specifications. Every few months, all cell lines were tested and authenticated using a panel of genetic and epigenetic markers.

Chromosome conformation capture

Chromosome conformation capture (3C) was performed to confirm direct interaction between the *PVT1* enhancer and the *MYC* promoter DNA sequences [[10](#_ENREF_10)]. In brief, chromatin fragments were fixed with formaldehyde. Following digestion with a restriction enzyme (EcoRI) and ligation using T4 ligase, PCR was performed to identify DNA sequences where parts of the *PVT1* and *MYC* had been ligated due to attachment between the enhancer and promoter. Primer sequences are shown in **Supplementary Table 5**.

Total RNA extraction and cDNA synthesis

Surgical specimens were homogenized with a Mixer Mill MM 300 homogenizer (Qiagen, Valencia, CA, USA). Total RNA from tissues and cell lines was isolated using miRNeasy Mini Kit (QIAGEN) according to the manufacturer’s instructions. cDNA was synthesized from 5.0 µg total RNA with High-Capacity cDNA Reverse Transcription Kit (Thermo Fisher Scientific, Waltham, MA, USA).

Nuclear and cytoplasmic fractionation of RNA from cancer cells

RNA was isolated from both nuclear and cytoplasmic fractions separately using the PARIS system (Thermo Fisher Scientific). The amount of the *PVT1* lncRNA was quantified in each fraction using U6 snRNA (nucleus-specific) and the GAPDH mRNA (cytoplasm-specific) as control.

Quantitative analysis of RNA expression

Quantitative real-time PCR (RT-qPCR) was performed using the StepOne Real Time PCR System and Fast SYBR Green Master Mix (Life Technologies, Waltham, MA, USA). β-actin was used as a normalization control. Relative expression of each mRNA was determined using the ΔΔCt method. Primer sequences are shown in **Supplementary Table 5**. RNA-seq was performed in three matched pair of CRC and adjacent normal mucosa to analyze transcriptional activity in chromosome 8q24.

*PVT1* knockdown by antisense oligonucleotides

Two types of antisense oligonucleotides (ASO) against the *PVT1* lncRNA and negative control ASO were synthesized by IDT (Integrated DNA Technologies, Coralville, Iowa, USA, **Supplementary Table 6**). Transfection of ASO (100 nM) was performed by the forward method according to the manufacturer’s protocol with a mixture of Optimem I (Invitrogen, Carlsbad, CA, USA) and Lipofectamine RNAiMAX Transfection Reagent (Thermo Fisher Scientific). Cells were incubated in culture media for 48 h after transfection prior to harvesting for analyses.

*PVT1* knockdown by BRD4 inhibitor JQ1

Cells were cultured in medium including DMSO or 1 µM JQ1 dissolved in DMSO for 24 h prior to harvesting for analyses. The *PVT1* knockdown levels were confirmed using RT-qPCR.

Western immunoblotting

Western immunoblotting experiments were performed as described previously [[11](#_ENREF_11)]. Anti-cMYC (sc-40, Santa Cruz Biotechnology, Dallas, TX USA, 1:250 dilution) antibody was used to detect cMYC protein, and anti-β-actin antibody (A5441, Sigma, St. Louis, MO, USA, 1:5000 dilution) was used to quantify the loading control.

MTT, invasion and migration assays

The MTT (3-(4, 5-dimethylthiazol-2-yl)-2, 5-diphenyltetrazolium bromide) assay (Sigma-Aldrich) was performed to measure proliferative activity, using a method described previously [[12](#_ENREF_12)]. The invasion and migratory ability of cancer cells were evaluated using BioCoat Matrigel Invasion Chambers (Corning Life Sciences, Tewksbury, MA, USA) as described previously [[12](#_ENREF_12)]. Briefly, 5×10^5^ cells were seeded into the invasion/migration chambers in serum-free medium with or without 1 µM of the BRD4 inhibitor JQ1. The cells that invaded the underside of the membrane were counted after 24 hours.

Methylation analysis

The methylation pattern of the *PVT1* locus was analyzed comprehensively using TCGA database [[4](#_ENREF_4)]. Additionally, the *PVT1* enhancer methylation was analyzed using pyrosequencing. Briefly, DNA derived from the clinical Cohort 3 was bisulfite modified by EZ DNA Methylation-Gold Kit (Zymo Research, Irvine, CA, USA) according to manufacturer’s instructions. After PCR of the target locus, the methylation rate was calculated using PyroMark Q96 MD (Qiagen). The primer set for the *PVT1* methylation analysis is shown in **Supplementary Table 5**.

ChIP assays for detecting binding of BRD4

The data of ChIP assays to detect binding of BRD4 was obtained from GEO database (GEO GSE73319)[[13](#_ENREF_13)].

BECC and Reactome pathway analyses

The detailed method is in **DETAILED SUPPLEMENTARY METHODS FOR PATHWAY ANALYSIS**.

Statistical analysis

Results are expressed as means ± standard errors (SE). JMP software (ver. 10.0, SAS Institute Inc., Cary, NC, USA) was used to perform the statistical analyses. The Wilcoxon’s rank sum test was used to compare continuous variables. Steel Dwass’ test was used for multiple comparisons. Overall survival (OS) was measured from the date of surgery to the date of cancer-related death. The Kaplan–Meier method with log rank correction was used to estimate distributions of OS in each patient group. In order to determine the cut-off value for the *PVT1* expression, we first established receiver operating characteristic (ROC) curves to discriminate the patients who did or did not survive. Youden’s index was used to determine the optimal cutoff threshold for the *PVT1* expression values from each cohort to predict the OS [[12](#_ENREF_12), [14-16](#_ENREF_14)]. Cox’s proportional hazard models were used to calculate hazard ratios (HR) with corresponding 95% confidence intervals (CI) for each group in a multivariate analysis. All calculated P values are two-sided, and a P value of <0.05 was considered to be statistically significant. We used standard error of mean (SEM) to derive error bars.

**Detailed Supplementary methods FOR PATHWAY ANALYSIS**

Data Collection and Annotation

Publicly available microarray and RNASeq databases were downloaded from the National Center for Biotechnology Information (NCBI) Gene Expression Omnibus (GEO) website [[17-19](#_ENREF_17)]. Gene expression summarization was performed by normalizing Affymetrix platforms by RMA (Robust Multichip Average) [[20](#_ENREF_20), [21](#_ENREF_21)] and RNASeq platforms by computing TPM (Transcripts Per Millions) [[22](#_ENREF_22), [23](#_ENREF_23)] values whenever normalized data were not available in GEO. We used log2(TPM) if TPM > 1 and (TPM – 1) if TPM < 1 as the final gene expression value for analyses.

BECC (Boolean Equivalent Correlated Clusters) Analysis

BECC analysis [[24](#_ENREF_24)] is based on Boolean Equivalent relationships, pair-wise correlation and linear regression analysis. BECC analysis begins with a seed gene. We used PVT1 as a seed gene in this paper. BECC analysis identified a set of genes Boolean Equivalent to PVT1 using the BooleanNet statistic (pertinent details are described below) on the TCGA COAD+READ (n = 521+177=698) dataset. A gene signature score is computed using the 67-genes that were equivalent to PVT1 which is used to order the sample.

Boolean Analysis

**Boolean logic** is a simple mathematic relationship of two values, i.e., high/low, 1/0, or positive/negative. The Boolean analysis of gene expression data requires the conversion of expression levels into two possible values. The ***StepMiner*** algorithm is reused to perform Boolean analysis of gene expression data [[25](#_ENREF_25)]. **The Boolean analysis** is a statistical approach which creates binary logical inferences that explain the relationships between phenomena. Boolean analysis is performed to determine the relationship between the expression levels of pairs of genes. The ***StepMiner*** algorithm is applied to gene expression levels to convert them into Boolean values (high and low). In this algorithm, first the expression values are sorted from low to high and a rising step function is fitted to the series to identify the threshold. Middle of the step is used as the StepMiner threshold. This threshold is used to convert gene expression values into Boolean values. A noise margin of 2-fold change is applied around the threshold to determine intermediate values, and these values are ignored during Boolean analysis. In a scatter plot, there are four possible quadrants based on Boolean values: (low, low), (low, high), (high, low), (high, high). A Boolean implication relationship is observed if any one of the four possible quadrants or two diagonally opposite quadrants are sparsely populated. Based on this rule, there are six kinds of Boolean implication relationships. Two of them are symmetric: equivalent (corresponding to the positively correlated genes), opposite (corresponding to the highly negatively correlated genes). Four of the Boolean relationships are asymmetric and each corresponds to one sparse quadrant: (low => low), (high => low), (low => high), (high => high). BooleanNet statistics is used to assess the sparsity of a quadrant and the significance of the Boolean implication relationships [[25](#_ENREF_25), [26](#_ENREF_26)]. Given a pair of genes A and B, four quadrants are identified by using the StepMiner thresholds on A and B by ignoring the Intermediate values defined by the noise margin of 2 fold change (+/- 0.5 around StepMiner threshold). Number of samples in each quadrant are defined as a_00_, a_01_, a_10_, and a_11_ which is different from X in the previous equation of F stat. Total number of samples where gene expression values for A and B are low is computed using the following equations.

${nA}_{low}= \left( a_{00}+ a_{01} \right), {nB}_{low}= \left( a_{00}+ a_{10} \right)$*,*

Total number of samples considered is computed using following equation.

$total= a_{00}+ a_{01}+ a_{10}+ a_{11}$

Expected number of samples in each quadrant is computed by assuming independence between A and B. For example, expected number of samples in the bottom left quadrant e_00_ = $\hat{n}$ is computed as probability of A low ((a_00_ + a_01_)/total) multiplied by probability of B low ((a_00_ + a_10_)/total) multiplied by total number of samples. Following equation is used to compute the expected number of samples.

$n= a_{ij}$*,* $\hat{n}= \left( {{nA}_{low}}/{total}* {{nB}_{low}}/{total} \right)*total$

To check whether a quadrant is sparse, a statistical test for (e_00_ > a_00_) or ($\hat{n}>n)$ is performed by computing S_00_ and p_00_ using following equations. A quadrant is considered sparse if S_00_ is high ($\hat{n}>n)$ and p_00_ is small.

$$S_{ij}= \frac{\hat{n}-n}{\sqrt{\hat{n}}}$$

$$p_{00}= \frac{1}{2} \left( \frac{a_{00}}{(a_{00}+ a_{01})}+ \frac{a_{00}}{(a_{00}+a_{10})} \right)$$

A suitable threshold is chosen for S_00_ > sThr and p_00_ < pThr to check sparse quadrant. A Boolean implication relationship is identified when a sparse quadrant is discovered using following equation.

***Boolean Implication*** = (*S_ij_* > sThr, *p_ij_* < pThr)

A relationship is called Boolean equivalent if top-left and bottom-right quadrants are sparse.

*Equivalent* $= \left( S_{01}> sThr, P_{01}< pThr, S_{10}> sThr, P_{10}< pThr \right)$

Boolean opposite relationships have sparse top-right (a_11_) and bottom-left (a_00_) quadrants.

*Opposite*$= \left( S_{00}> sThr, P_{00}< pThr, S_{11}> sThr, P_{11}< pThr \right)$

Boolean equivalent and opposite are symmetric relationship because the relationship from A to B is same as from B to A. Asymmetric relationship forms when there is only one quadrant sparse (A low => B low: top-left; A low => B high: bottom-left; A high=> B high: bottom-right; A high => B low: top-right). These relationships are asymmetric because the relationship from A to B is different from B to A. For example, A low => B low and B low => A low are two different relationships.

A low => B high is discovered if the bottom-left (a_00_) quadrant is sparse and this relationship satisfies following conditions.

*A low => B high* = ($S_{00}> sThr, P_{00}< pThr$)

Similarly, A low => B low is identified if the top-left (a_01_) quadrant is sparse.

*A low => B low* = ($S_{01}> sThr, P_{01}< pThr$)

A high => B high Boolean implication is established if the bottom-right (a_10_) quadrant is sparse as described below.

*A high => B high* = ($S_{10}> sThr, P_{10}< pThr$)

Boolean implication A high => B low is found if the top-right (a_11_) quadrant is sparse using following equation.

*A high => B low* = ($S_{11}> sThr, P_{11}< pThr$)

For each quadrant a statistic S_ij_ and an error rate p_ij_ is computed. S_ij_ > sThr and p_ij_ < pThr are the thresholds used on the BooleanNet statistics to identify Boolean implication relationships.

Boolean analyses in the TCGA dataset uses a threshold of sThr = 5 and pThr = 0.18. Compared to previously used thresholds sThr = 3 and pThr = 0.1 for BooleanNet [[24](#_ENREF_24), [25](#_ENREF_25), [27](#_ENREF_27)], we increased the sThr and relax the pThr to focus on reasonable number of candidate genes. False discovery rate of Boolean Equivalent relationships identification using sThr = 5 and pThr = 0.18 on the TCGA COAD+READ dataset is less than 0.0001 (close to zero).

Statistical Analyses

Gene signature is used to classify sample categories and the performance of the multi-class classification is measured by ROC-AUC (Receiver Operating Characteristics Area Under The Curve) values. A color-coded bar plot is combined with a density plot to visualize the gene signature-based classification. All statistical tests were performed using R version 3.2.3 (2015-12-10). Standard t-tests were performed using python scipy.stats.ttest_ind package (version 0.19.0) with Welch’s Two Sample t-test (unpaired, unequal variance (equal_var=False), and unequal sample size) parameters. Multiple hypothesis correction were performed by adjusting *p* values with statsmodels.stats.multitest.multipletests (fdr_bh: Benjamini/Hochberg principles). The results were independently validated with R statistical software (R version 3.6.1; 2019-07-05). Pathway analysis of gene lists were carried out via the Reactome database and algorithm [[28](#_ENREF_28)]. Reactome identifies signaling and metabolic molecules and organizes their relations into biological pathways and processes. Kaplan-Meier analysis is performed using lifelines python package version 0.14.6 (**Supplementary Table 7**).

**REFERENCES**

1. cBioPortal: [*http://wwwcbioportalorg/indexdo*](http://wwwcbioportalorg/indexdo) Accessed in December 1, 2014.

2. Gao J, Aksoy BA, Dogrusoz U, Dresdner G, Gross B, Sumer SO, Sun Y, Jacobsen A, Sinha R, Larsson E, et al: **Integrative analysis of complex cancer genomics and clinical profiles using the cBioPortal.** *Sci Signal* 2013, **6:**pl1.

3. Cerami E, Gao J, Dogrusoz U, Gross BE, Sumer SO, Aksoy BA, Jacobsen A, Byrne CJ, Heuer ML, Larsson E, et al: **The cBio cancer genomics portal: an open platform for exploring multidimensional cancer genomics data.** *Cancer Discov* 2012, **2:**401-404.

4. TCGA_Research_Network: [*http://cancergenomenihgov/*](http://cancergenomenihgov/) Accessed in May1, 2014.

5. Andersson R, Gebhard C, Miguel-Escalada I, Hoof I, Bornholdt J, Boyd M, Chen Y, Zhao X, Schmidl C, Suzuki T, et al: **An atlas of active enhancers across human cell types and tissues.** *Nature* 2014, **507:**455-461.

6. FANTOM5: [*http://fantomgscrikenjp/*](http://fantomgscrikenjp/) Accessed in July 1, 2015.

7. FANTOM5_Human_Enhancer_Tracks: [*http://slidebasebinfkudk/human_enhancers/presets*](http://slidebasebinfkudk/human_enhancers/presets) Accessed in December 2, 2019.

8. UCSC_Genome_Browser: [*http://genomeucscedu/*](http://genomeucscedu/) Accessed in June 1, 2015.

9. Kent WJ, Sugnet CW, Furey TS, Roskin KM, Pringle TH, Zahler AM, Haussler D: **The human genome browser at UCSC.** *Genome Res* 2002, **12:**996-1006.

10. Cope NF, Fraser P: **Chromosome conformation capture.** *Cold Spring Harb Protoc* 2009, **2009:**pdb prot5137.

11. Toden S, Okugawa Y, Buhrmann C, Nattamai D, Anguiano E, Baldwin N, Shakibaei M, Boland CR, Goel A: **Novel Evidence for Curcumin and Boswellic Acid-Induced Chemoprevention through Regulation of miR-34a and miR-27a in Colorectal Cancer.** *Cancer Prev Res (Phila)* 2015, **8:**431-443.

12. Okugawa Y, Toiyama Y, Hur K, Toden S, Saigusa S, Tanaka K, Inoue Y, Mohri Y, Kusunoki M, Boland CR, Goel A: **Metastasis-associated long non-coding RNA drives gastric cancer development and promotes peritoneal metastasis.** *Carcinogenesis* 2014, **35:**2731-2739.

13. McCleland ML, Mesh K, Lorenzana E, Chopra VS, Segal E, Watanabe C, Haley B, Mayba O, Yaylaoglu M, Gnad F, Firestein R: **CCAT1 is an enhancer-templated RNA that predicts BET sensitivity in colorectal cancer.** *J Clin Invest* 2016, **126:**639-652.

14. Tanaka S, Hattori N, Ishikawa N, Shoda H, Takano A, Nishino R, Okada M, Arihiro K, Inai K, Hamada H, et al: **Krebs von den Lungen-6 (KL-6) is a prognostic biomarker in patients with surgically resected nonsmall cell lung cancer.** *Int J Cancer* 2012, **130:**377-387.

15. Toiyama Y, Takahashi M, Hur K, Nagasaka T, Tanaka K, Inoue Y, Kusunoki M, Boland CR, Goel A: **Serum miR-21 as a diagnostic and prognostic biomarker in colorectal cancer.** *J Natl Cancer Inst* 2013, **105:**849-859.

16. Hur K, Toiyama Y, Schetter AJ, Okugawa Y, Harris CC, Boland CR, Goel A: **Identification of a metastasis-specific MicroRNA signature in human colorectal cancer.** *J Natl Cancer Inst* 2015, **107**.

17. Barrett T, Suzek TO, Troup DB, Wilhite SE, Ngau WC, Ledoux P, Rudnev D, Lash AE, Fujibuchi W, Edgar R: **NCBI GEO: mining millions of expression profiles--database and tools.** *Nucleic Acids Res* 2005, **33:**D562-566.

18. Barrett T, Wilhite SE, Ledoux P, Evangelista C, Kim IF, Tomashevsky M, Marshall KA, Phillippy KH, Sherman PM, Holko M, et al: **NCBI GEO: archive for functional genomics data sets--update.** *Nucleic Acids Res* 2013, **41:**D991-995.

19. Edgar R, Domrachev M, Lash AE: **Gene Expression Omnibus: NCBI gene expression and hybridization array data repository.** *Nucleic Acids Res* 2002, **30:**207-210.

20. Irizarry RA, Bolstad BM, Collin F, Cope LM, Hobbs B, Speed TP: **Summaries of Affymetrix GeneChip probe level data.** *Nucleic Acids Res* 2003, **31:**e15.

21. Irizarry RA, Hobbs B, Collin F, Beazer-Barclay YD, Antonellis KJ, Scherf U, Speed TP: **Exploration, normalization, and summaries of high density oligonucleotide array probe level data.** *Biostatistics* 2003, **4:**249-264.

22. Li B, Dewey CN: **RSEM: accurate transcript quantification from RNA-Seq data with or without a reference genome.** *BMC Bioinformatics* 2011, **12:**323.

23. Pachter L: **Models for transcript quantification from RNA-Seq.** In *arXiv e-prints*2011.

24. Dabydeen SA, Desai A, Sahoo D: **Unbiased Boolean analysis of public gene expression data for cell cycle gene identification.** *Mol Biol Cell* 2019, **30:**1770-1779.

25. Sahoo D, Dill DL, Gentles AJ, Tibshirani R, Plevritis SK: **Boolean implication networks derived from large scale, whole genome microarray datasets.** *Genome Biol* 2008, **9:**R157.

26. Sahoo D, Seita J, Bhattacharya D, Inlay MA, Weissman IL, Plevritis SK, Dill DL: **MiDReG: a method of mining developmentally regulated genes using Boolean implications.** *Proc Natl Acad Sci U S A* 2010, **107:**5732-5737.

27. Pandey S, Sahoo D: **Identification of gene expression logical invariants in Arabidopsis.** *Plant Direct* 2019, **3:**e00123.

28. Fabregat A, Jupe S, Matthews L, Sidiropoulos K, Gillespie M, Garapati P, Haw R, Jassal B, Korninger F, May B, et al: **The Reactome Pathway Knowledgebase.** *Nucleic Acids Res* 2018, **46:**D649-D655.
